# SL-BioDP: Multi-Cancer Interactive Tool for Prediction of Synthetic Lethality and Response to Cancer Treatment

**DOI:** 10.3390/cancers11111682

**Published:** 2019-10-29

**Authors:** Xiang Deng, Shaoli Das, Kristin Valdez, Kevin Camphausen, Uma Shankavaram

**Affiliations:** 1 Bioinformatics core facility, Radiation Oncology Branch, National Cancer Institute, National Institutes of Health, Bethesda, MD 20892, USA; dengx@mail.nih.gov (X.D.); shaoli.das@nih.gov (S.D.); kristin.valdez@nih.gov (K.V.); camphauk@mail.nih.gov (K.C.); 2 Frederick National Laboratory for Cancer Research, Leidos Biomedical Research, Inc., Frederick, MD 21702, USA

**Keywords:** cancer, synthetic lethality, web application, bioinformatics, DNA repair pathway

## Abstract

Synthetic lethality exploits the phenomenon that a mutation in a cancer gene is often associated with new vulnerability which can be uniquely targeted therapeutically, leading to a significant increase in favorable outcome. DNA damage and survival pathways are among the most commonly mutated networks in human cancers. Recent data suggest that synthetic lethal interactions between a tumor defect and a DNA repair pathway can be used to preferentially kill tumor cells. We recently published a method, DiscoverSL, using multi-omic cancer data, that can predict synthetic lethal interactions of potential clinical relevance. Here, we apply the generality of our models in a comprehensive web tool called Synthetic Lethality Bio Discovery Portal (SL-BioDP) and extend the cancer types to 18 cancer genome atlas cohorts. SL-BioDP enables a data-driven computational approach to predict synthetic lethal interactions from hallmark cancer pathways by mining cancer’s genomic and chemical interactions. Our tool provides queries and visualizations for exploring potentially targetable synthetic lethal interactions, shows Kaplan–Meier plots of clinical relevance, and provides in silico validation using short hairpin RNA (shRNA) and drug efficacy data. Our method would thus shed light on mechanisms of synthetic lethal interactions and lead to the discovery of novel anticancer drugs.

## 1. Introduction

Drug treatment of cancer depends on the notion that mutations that give rise to the development of cancer also bring about a weakness that can be exploited therapeutically. Large-scale cancer genome sequencing efforts have catalogued mutations in various cancer types that can be explored as tumor-specific vulnerabilities [1]. These genetic alterations consist of gain-of-function mutations in which genes are amplified, translocated, or mutated and loss-of-function mutations in which gene function is compromised by missense mutations or deletions. The former group of mutations have been the subject of intense focus by the pharmaceutical industry for the development of targeted cancer drugs. These efforts have resulted in several cancer drugs that target activated driver oncogenes, such as *HER2*, *BCR-ABL*, *EGFR*, and *BRAF* [2]. These drugs target signaling proteins that are aberrantly activated as a direct consequence of an oncogenic mutation, and hence their inhibition is detrimental to the cancers. This dependence on oncogenic driver pathways is commonly referred to as oncogene addiction [3]. From a drug discovery perspective, the loss-of-function mutations are much harder to tackle, and the same is true for several activated oncogenes that have proven to be undruggable, such as the MYC transcription factor and the RAS proteins. Therefore, alternative strategies are needed to target the vulnerabilities induced by these classes of cancer-causing genes. 

One promising way to tackle this challenge is based on the concept of synthetic lethality (SL). SL describes the relationship between two genes whereby inactivation of either gene alone results in a viable phenotype, while their combined inactivation is lethal. SL has long been considered a foundation for the development of selective anticancer therapies [4,5,6,7,8], which aim to inhibit the SL partner of a gene that is inactivated de novo in the cancer cells. Beyond guiding the development of novel selective cancer therapies, it has been noted that the network of SL interactions can give a bird’s eye view of the genomic state of a given tumor that can be used to find tumor-specific vulnerabilities and develop effective synergistic drug combination therapies in a precision-based manner [9,10].

Given the importance of SL, considerable work has been devoted to finding such interactions in cancer—both experimentally [4,5,6,7,8] and computationally [11,12]. Nevertheless, so far, the utility of SL in the clinic has been limited, and many of the SLs found in current screens manifest a poor predictive signal in actual patients’ data. A recent publication tried to bridge this gap by finding the clinically relevant SL interactions from cell-line based SL screens [13]. But again, cell-line based SL screens have been done for only a limited set of cancer genes and cell lines. So, there is a need for a comprehensive resource that can be queried for alternative drug targets for important cancer genes based on the concept of synthetic lethality, which shows potential to be clinically relevant. A previously published database, namely, SynLethDB [14] has a collection of synthetic lethal partners from multiple sources (text mining, synthetic lethality screens, and computational prediction), but this database lacks the parameters to assess the clinical relevance of these SL interactions. Here, we present an integrative web portal, Synthetic Lethality BioDiscovery Portal (SL-BioDP), that enables multilevel querying and visualization of synthetic lethality-based potential drug targets for genes which are frequently mutated in cancers. To explore the possibilities of precision therapies for specific gene alterations using the concept of synthetic lethality, we inferred SL interactions of known cancer-driver genes or hallmark cancer pathways. A published statistical approach [15] was used to identify potential SL interaction from 18 different cancers from the TCGA cohort (The Cancer Genome Atlas) in a genome-scale manner, and assess their clinical relevance. Combined with in silico validation from large-scale in vitro drug response and shRNA screens, our resource gives a venue to query and visualize drug targets, and allows hypothesis generation for personalized medicine.

## 2. Results

### 2.1. Data Summary

**Figure 1:**
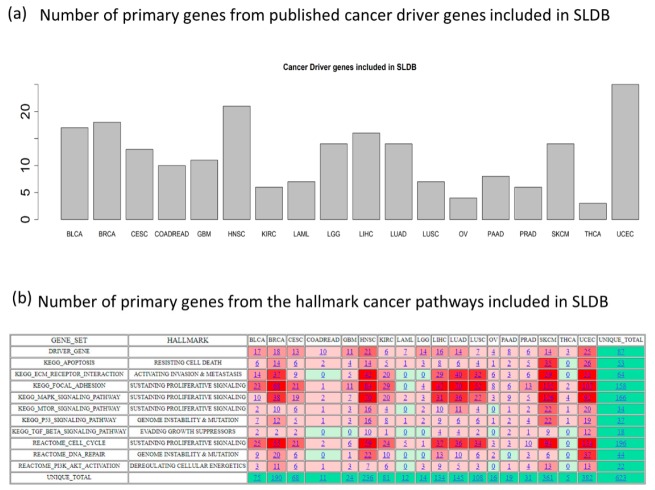
General schema of SL-BioDP: (**a**) The bar-chart shows the number of cancer driver genes in 18 cancer types included as primary genes in SL-BioDP; (**b**) the heatmap shows the number of genes from each of the 10 hallmark cancer pathways included as primary genes in SL-BioDP for each of the 18 cancer types. The color coding is based on the frequency of the number of genes from a pathway in each cancer (red: more number of genes, green: less number of genes).

SL-BioDP predicts synthetic lethal partners of reported cancer driver genes and genes from hallmark cancer pathways in 18 cancer types. Cancers are mainly caused by mutations/alterations of specific genes or pathways that contribute to the initiation and progression of the disease. In our study, we aimed to find potentially targetable and clinically relevant synthetic lethal partners for reported cancer driver genes [16] and genes belonging to 10 hallmark cancer pathways [17] in 18 types of cancers from TCGA (as shown in Figure 1a,b). For the synthetic lethality prediction model in each cancer type, we only included genes which are mutated in at least five tumor samples in TCGA. Figure 1a,b shows the number of mutated driver genes and the number of genes from 10 cancer pathways included in each of the 18 cancer types, which totals 623 unique primary genes. The synthetic lethal partners for the primary genes in the cancer types are predicted using DiscoverSL [15]. For cancer-specific synthetic lethal interactions of each of the 623 primary genes reported in SL-BioDP, the DiscoverSL algorithm was run on the whole genome-scale somatic mutation, expression, copy-number alteration, and clinical data of 7654 tumor samples from 18 TCGA cancer types. Published reports of interactions from SL-screening data were collected from two sources [13,14] and overlapped with the predictions from DiscoverSL. Table 1 shows the number of SL interactions for each cancer type either supported by Achilles short hairpin RNA (shRNA) screenings or from literature. Outside the database, we tested an additional in silico validation from CRISPR (clustered regularly interspaced short palindromic repeats) knockout data from project Achilles (DepMap Public 19Q3) on 12 selected cancer driver genes frequently mutated in cancers: *BRCA1*, *BRCA2*, *TP53*, *PTEN*, *ATM*, *ATR*, *KRAS*, *HRAS*, *BRAF*, *EGFR*, *MET*, and *PIK3CA*. Many of the predicted SL interactions of these 12 genes across 18 cancers were also present in the genetic dependency CRISPR screen (shown in Supplementary Table S1). A pathway enrichment analysis for SL partners for a primary gene in a cancer type can be viewed. Finally, drugs targeting the synthetic lethal partners can be explored for a chosen list of genes. For SL-based drug target searching, gene–drug interactions from the two databases, DrugBank [18] and DGIdb [19], were incorporated which total 20,899 and 28,104 gene–drug interactions, respectively.

**Figure 2:**
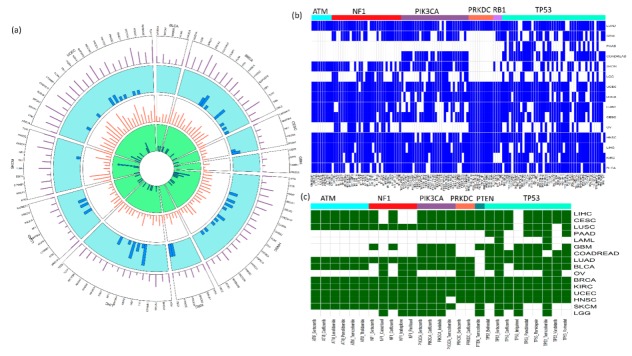
Summarization of cancer-specific synthetic lethality data presented in SL-BioDP: (**a**) Circos plots displaying the summary of synthetic lethal interactions of 63 cancer genes that are common in at least five cancer types. The outermost layer (layer 1) shows the number of predicted synthetic lethals per gene. The next layer (layer 2) shows histograms of the number of mutations per gene. The next to last layer (layer 3) shows the number of drugs identified per gene, through their synthetic lethal partners. Finally, the innermost layer (layer 4) displays histograms of the number of enriched synthetic lethal pathways per gene; (**b**) the table shows all common synthetic lethal pairs with high prediction scores (>0.7) in at least 10 cancer types. For each synthetic lethal pair (columns) and each cancer type (row), the color coding shows whether that SL pair has a high prediction score (>0.7) in that cancer type (color: blue) or not (color: white). The SL pairs in columns are grouped by their primary genes (*ATM*, *NF1*, *PIK3CA*, *PRKDC*, *RB1*, and *TP53*); (**c**) similar to the table in (**b**), this table shows the common SL-based drugs targeting the common SL partners of the primary genes (*ATM*, *NF1*, *PIK3CA*, *PRKDC*, *PTEN*, and *TP53*) in at least 10 cancer types.

Summary of the data included in SL-BioDP is shown in Figure 2. The circos plot in Figure 2a shows an example of selected primary genes common in multiple cancer types. Each sector in the circos plot corresponds to a cancer type and the x-axis labels show the primary genes included in the cancer type. For each primary gene in a cancer type, information on the number of predicted synthetic lethal genes, number of mutated samples, number of drugs targeting synthetic lethal genes, and number of enriched synthetic lethal pathways are shown in tracks 1, 2, 3, and 4, respectively (see Supplementary Tables S2 and S3). Depending on the gene expression, copy number, and mutation profiles of the genes in each cancer types, synthetic lethality of gene pairs can be cancer-specific. However, we found a common synthetic lethal signature in multiple cancers for some very commonly mutated cancer genes: *ATM*, *NF1*, *PIK3CA*, *PRKDC*, *RB1*, and *TP53*. Figure 2b shows a map of common synthetic lethal pairs (common in at least 10 cancer types) in cancers, grouped by the primary genes. To support the idea of precision therapeutic approaches for treating cancer patients having specific genetic alterations, we demonstrate the utility of SL-BioDP for suggesting SL-based drugs depending on the presence of mutations in specific cancer genes. From the SL partners of frequently mutated cancer driver genes, we inferred the common drugs (used for cancer treatment in the National Cancer Institute (NCI)) targeting these SL partners from DrugBank and DGIdb. These drugs can be potential treatment choices for treating patients carrying mutations in their corresponding primary genes in multiple types of cancers (Figure 2c).

### 2.2. Searchability and Browsing

**Figure 3:**
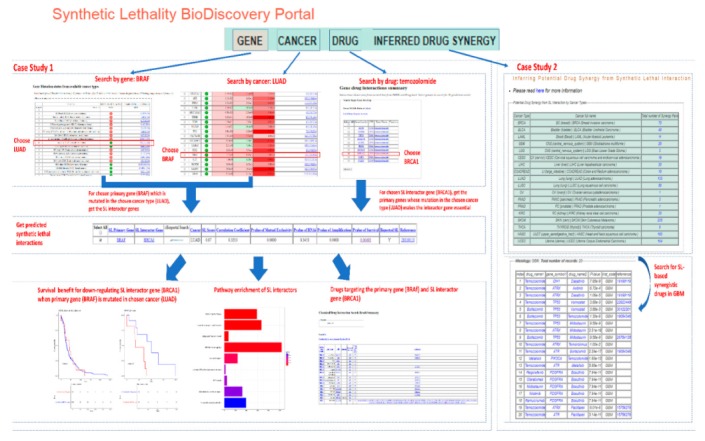
The utility of SL-BioDP is shown with a case study on *BRAF* in cancer lung adenocarcinoma (LUAD). Using “GENE” search in SL-BioDP, the mutation and expression alteration profile of *BRAF* across 18 tumor histology types is shown. *BRAF* is commonly mutated in LUAD (4.26%). Otherwise, using the “CANCER” search in SL-BioDP and choosing LUAD as the histology, genes having the most significant alteration in expression (comparing tumor vs. normal samples from TCGA) are displayed. *BRAF* shows more than 6-fold upregulation in tumor samples (*p*-value < 0.001). When searched for SL interactions, *BRCA1* is a predicted SL partner of *BRAF* in LUAD (synthetic lethal score = 0.87 and survival *p*-value = 0.0049). Alternatively, using “DRUG” search in SL-BioDP, searching for the targets of the drug “temozolomide” yields 10 target genes. By selecting the *BRCA1* gene and hitting submit, we search for the alteration profiles of *BRCA1* across 18 cancers. From the resulting page we can select the histology type (we choose LUAD here) to search for the primary genes whose mutation makes *BRCA1* synthetic lethal in the selected cancer type. Restricting the search for reported SL interactions only, we get primary (mutated) genes including *BRAF*. Survival analysis (Kaplan-Meier plot) shows better disease-free survival in patients with underexpression of *BRCA1* compared to overexpression, in LUAD samples with *BRAF* mutations. Pathway enrichment for the SL interactors of the primary gene (*BRAF*) can be seen. The drugs targeting either of the SL gene pair is searchable from DGIdb or DrugBank by selection.

Users can search SL-BioDP from any of the three modes: (1) “GENES” tab: using a primary gene of interest (the model currently includes 623 cancer genes commonly mutated in cancers), (2) “CANCER” tab: tumor tissue of origin (currently includes 18 cancer histology types), and (3) “DRUG” tab: drugs of interest (drug targets are compiled from the two databases, DrugBank and DGIdb). Additionally, potential synergistic drug combinations for different cancers can be browsed from the “INFERRED DRUG SYNERGY” tab. An example of search and browsing functionalities in SL-BioDP is illustrated in Figure 3.

**Figure 4:**
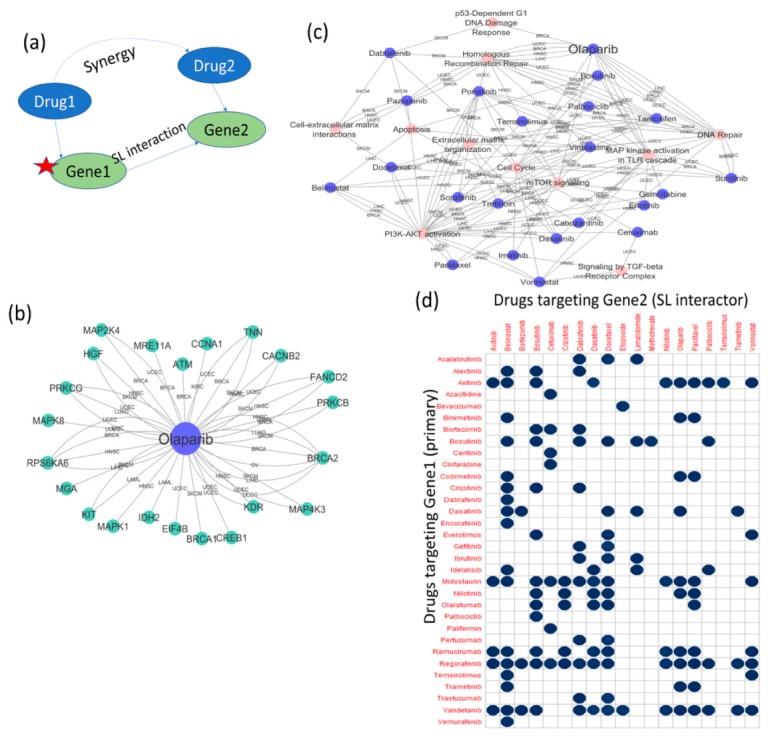
Mutations in cancer genes make cancer cells sensitive to certain drugs. (**a**) A schematic diagram showing the concept of potential synergy between drugs targeting synthetic lethal partners. If Gene2 is a predicted synthetic lethal partner of Gene1, and a mutation in Gene1 makes cancer cell lines sensitive to a drug targeting Gene2 (Drug2), then a drug targeting Gene1 (Drug1) may have synergy with the drug targeting Gene2 (Drug2); (**b**) combining our SL predictions with analysis of drug sensitivity data in cancer cell lines, the PARP inhibitor drug olaparib was identified to be sensitive to the presence of mutations in primary genes in multiple cancer types. Apart from *BRCA1*/*2*, sensitivity to mutations in *ATM*, *FANCD2*, *MRE11A*, *CCNA1*, *CACNB2*, *PRKCB*, *PRKCG*, *MAPK1*, *MAPK8*, *MAP2K4*, *MAP4K3*, *CREB1*, *IDH1*, *KDR*, *KIT*, *EIF4B*, *HGF*, *MGA*, and *RPS6KA6* was identified; (**c**) mutated pathway–drug network shows the enriched pathways constituting of mutated primary genes that make cancer cell lines sensitive to particular drugs. This mutated pathway enrichment study explains the known phenomenon that certain drugs like olaparib are sensitive to deficiency in homologous recombination repair, and also points toward additional use cases for drugs (e.g., olaparib) in tumors having alterations in other known cancer pathways. For example, olaparib also shows sensitivity to the alteration of MAPK signaling, mTOR signaling, and p53-dependent DNA damage response; (**d**) potential drug synergy matrix in cancer: *BRCA*, derived from predicted SL interactions from SL-BioDP, and conditional drug sensitivity calculation from GDSC drug screening data (measuring whether the presence of a mutation in the primary gene or Gene1 makes cancer cell lines sensitive to drugs targeting Gene2). Drugs targeting predicted SL interactors with significantly greater sensitivity in the presence of primary gene mutations (*p* < 0.05) are plotted along the *y*-axis, while drugs targeting the primary genes, whose mutations make cancer cell lines sensitive to the drugs in the *y*-axis are plotted along the *x*-axis. The blue dots in any cell of the matrix represent that there may be a potential synergy between the corresponding two drugs, as loss of function of the gene targeted by the drug in *x*-axis makes cancer cell lines sensitive to the drug in *y*-axis.

(1) Search using “GENE” tab: search can be initiated by using either the official gene symbol or Entrez gene id of the primary gene. SL-BioDP presents cancer-specific synthetic lethality information in multiple levels. First, the mutation and expression profiles of the primary genes in each cancer type are presented. Then, the predicted synthetic lethal partners of the chosen primary genes in selected cancer types are presented. The predicted synthetic lethal pairs in SL-BioDP are ranked by the synthetic lethal score calculated by the DiscoverSL model. Additional parameters for assessment of synthetic lethality for each predicted SL pair are shown as: a *p*-value for expression correlation, mutual exclusivity of mutations, conditional gene essentiality from RNAi screens, and a change in the SL interactor amplification profile in the presence of a primary gene mutation. For validation purposes, to show conditional gene essentiality, we provided an in silico validation approach using shRNA screening data from cancer cell lines [15]. Assessment of the clinical relevance of the predicted SL interactions are shown as Kaplan–Meier survival analyses for disease-free survival and for downregulation vs. upregulation of the predicted synthetic lethal interactor in clinical samples carrying the mutation in the primary gene. In the KM plots, the statistically significant cases (*p* < 0.05) where downregulation of the synthetic lethal interactor gene shows better disease-free survival in the presence of the mutation in the primary gene are ideal cases of clinically relevant synthetic lethality. Together with the amplification status of the SL interactor gene, we can hypothesize if a favorable clinical outcome can be seen by selective targeting of the interactor gene (Gene2). In addition, we provide reported SL interactions and supporting references of our findings whenever it is feasible.

(2) Search using “CANCER” tab: users can select from a drop-down list containing 18 tumor histology types. By selecting a particular histology, users are shown the top genes which are differentially expressed between the tumor vs. adjacent normal tissues from that histology. Then by selecting one or multiple gene(s) of interest, users can see their predicted SL partners, view enriched pathways, and search for drugs targeting the SL partners (as described above).

(3) Search using “DRUG” tab: users can search by the name of a drug of their interest and choose from any of the two databases, “DrugBank” or “DGIdb”, to see their targets. From the resulting drug-target page, the users can select one or multiple target gene(s) to search the primary genes for which the chosen target genes are SL partners. Or in other words, the primary genes which when mutated can be potentially treated with the drugs of interest, by targeting the SL partner genes.

(4) Browsing the “INFERRED DRUG SYNERGY” tab: users can browse for the potential synergistic drug combinations in the 18 cancer types. The inferred drug synergy table for each cancer type shows the potential synergistic drugs based on the predicted SL interactions of a primary mutated gene. The tables also include published references (if any) for the inferred synergistic drug combinations. An interesting inference coming from the gene mutation-based drug sensitivity study is the potential synergy between drugs targeting the primary gene (mutated) and the SL interactor gene. The potential synergy between two drugs targeting the primary gene and the SL interactor gene is inferred from this hypothesis: if the loss of function (presence of the mutation) of the primary gene (Gene1) makes cancer cell lines sensitive to certain drugs targeting the SL interactor gene(s), then it is possible that drugs that target the primary gene(s) will make cancer cells sensitive to the drugs that target the SL interactor gene(s). Information for drugs targeting the primary gene (Gene1) and the SL interactor gene (Gene2) are collected from the two databases, DrugBank and DGIdb. Gene1 and Gene2 SL interaction predictions are taken from SL-BioDP. Drug2 (targeting Gene2) sensitivity (*p* < 0.05) in cancer cells carrying the mutation in Gene1 (compared to cells not carrying the mutation in Gene1) is calculated from GDSC (Genomics of drug sensitivity in cancer) portal drug screening data (as explained above and in the methods section). In our database portal SL-BioDP, the potential synergy between cancer drugs in different cancer types, inferred from the predicted synthetic lethal interaction between their targets, can be explored in the tab “Inferred Drug Synergy”. The reasoning behind potential drug synergy is explained in Figure 4a, Drug1 (targeting Gene1) and Drug2 (targeting Gene2) are shown to be potentially synergistic if Gene1 and Gene2 are predicted synthetic lethal interactors and Drug2 shows conditional sensitivity in the presence of the mutation in Gene1. Figure 4b shows examples of potential drug synergy derived from SL interactions in four cancers: lung adenocarcinoma (LUAD), breast invasive carcinoma (BRCA), brain lower grade glioma (LGG), and glioblastoma multiforme (GBM). For inferring the synergy between cancer drugs, we only used the cancer drugs currently in use by NCI. Supplementary Table S4 shows the information of the potential synergy between NCI-approved drugs in different cancer types that has been supported by references in published literature or clinical trials (if the drug synergy is previously reported).

### 2.3. Case Study on Targetable SL Interactions and Drugs for BRAF Mutation in Lung Adenocarcinoma

The utility of SL-BioDP is shown with a case study on the primary gene *BRAF* in cancer lung adenocarcinoma (LUAD), a form of non-small-cell lung cancer (Figure 3). *BRAF* mutations are signature genetic alterations present in LUAD [20]. Two therapeutic agents for targeting *BRAF* in metastatic melanoma have been approved by the FDA; but a majority of patients are likely to develop drug resistance. In non-small-cell lung cancers, *BRAF* inhibitors are also shown to have a positive response [21]. Searching for the primary gene “*BRAF*” from the “GENES” tab in SL-BioDP shows the alteration profiles of *BRAF* in 18 different tumor types, both in terms of mutations and gene expression. *BRAF* is commonly mutated in some cancers, including 4.26% of the TCGA tumor samples with LUAD histology. While searching for genes commonly altered in LUAD from the “CANCER” tab in SL-BioDP, it shows that the gene *BRAF* is upregulated in LUAD tumor samples (logFC > 6, *p*-value < 0.001), compared to solid tissue normal samples in TCGA tumor sample data. By searching for the predicted mutation-based synthetic lethal targets of *BRAF* in LUAD (either by selecting the “LUAD” histology from the “GENE” search results page or by selecting “*BRAF*” from the “CANCER” search results page), we get predicted synthetic lethal interactors. *BRCA1* is a predicted SL partner of *BRAF* in LUAD (synthetic lethal score = 0.87 and survival *p*-value = 0.0049), which was also reported in published SL screens. Alternately, when a search for a drug target is performed using the “DRUG” tab in SL-BioDP, e.g., for the drug temozolomide, we first get its targets from one of the databases, DrugBank or DGIdb (chosen by the user). Then, the user can select one or multiple target genes of the drug to search for the primary (mutated) genes across the 18 tumor histology types in SL-BioDP. From this page, one or more histology type(s) can be selected to search for gene alteration patterns (e.g., *BRAF* mutations) in the chosen histology (e.g., LUAD) which can be potentially treated by the drug of interest (e.g., temozolomide) using a synthetic lethal approach, that is by targeting the synthetic lethal partner (e.g., *BRCA1*). Survival analysis (Kaplan–Meier plot) shows better disease-free survival in patients with underexpression of *BRCA1* compared to overexpression, in LUAD samples with *BRAF* mutations. Pathway enrichment for the SL interactors of the primary gene (*BRAF*) can be seen. Next, drug targets for selected SL interactors can be seen by searching either of the two databases: DGIdb or DrugBank. A search for drugs for the SL pair *BRAF*–*BRCA1* from DGIdb shows drugs targetable for either the SL gene, *BRCA1* or the primary gene, *BRAF*.

### 2.4. Utility Study on Applicability of the PARP-Inhibitor Drug Olaparib and Its Potential Synergy with Other Drugs

The main usefulness of the concept of synthetic lethality lies in finding alternative targets for cases with mutations/alterations in specific cancer genes or pathways. The use of PARP (poly ADP ribose polymerase) inhibitor drugs for treating breast cancers or ovarian cancers with *BRCA1*/*BRCA2* mutations is a widely documented example where synthetic lethality is used for effective treatment of cases with driver alterations [6,22]. PARP inhibitors have been reported to work for defects in homologous recombination repair of DNA double-strand breaks. We inferred potential synergistic combinations for the PARP inhibitor drug, olaparib. Figure 4a illustrates the concept behind the inferred drug synergy. In order to identify effective drug treatment strategies for alterations in specific cancer genes, we harnessed the predicted synthetic lethal interaction information from SL-BioDP, collected the drugs reported to target the predicted synthetic lethal genes from DrugBank or DGIdb (can be browsed from SL-BioDP), and then used cancer cell line drug screening data from the GDSC portal to predict sensitivity to the drugs targeting the synthetic lethal partners selectively in cells carrying mutations in the primary gene. The calculation of drug sensitivity is described in the methods section. Figure 4b shows the network of primary (altered) genes and drugs where the alterations in the primary genes made cancer cell lines sensitive to the drug, olaparib (also in Supplementary Table S5). The edges, connecting the primary genes and the drug, represent the cancer types where these potential treatment strategies can be used. The reported cases of *BRCA1* and *BRCA2* mutations that can be treated by PARP inhibitors are identified from this study. Also, there are reports on the use of PARP inhibitors for treating tumors with mutations in the gene *ATM* [23]. Our study shows that along with these validated examples there are other cases with alterations in driver genes, e.g., genes from the MAP kinase (Mitogen-activated protein kinase) pathways (*MAPK1*, *MAPK8*, *MAP2K4*, and *MAP4K3*), that can be potentially targeted by the PARP inhibitor, olaparib. When we performed pathway enrichment analysis on all the primary genes for which we detected sensitivity to the drugs (from the network in Figure 4b and Supplementary Table S6), as expected, we can see that olaparib is sensitive to the alteration of homologous recombination repair (Figure 4c). Interestingly, from this analysis we also identified other potentially important connections like alterations in the MAPK pathway and mTOR (mammalian target of rapamycin) pathway with olaparib. Through the “INFERRED DRUG SYNERGY” tab, searching for potential drug synergies in breast cancer (BRCA) (inferred from synthetic lethality and a mutation-specific drug sensitivity analysis as described previously), we identified that the drugs cobimetinib, binimetinib, trametinib, and temozolomide are potentially synergistic with olaparib (Figure 4d). Three of the potentially synergistic drugs (cobimetinib, binimetinib, and trametinib) are MEK inhibitors. A rational combination of MEK and PARP inhibitors have been reported to induce synergistic effects in multiple RAS mutant tumor models [24].

## 3. Discussion

Synthetic lethality has immense potential in cancer therapeutics. Where the driver genes or oncogenes cannot be targeted, synthetic lethal interactors can potentially serve as drug targets in the presence of mutations in the driver genes, as the mutant tumor cells are dependent upon the synthetic lethal interactors for their survival. Recent advances in RNAi and CRISPR technologies have enabled large-scale synthetic lethality screens for individual genes to be performed in human cell lines. The genetic dependency map (DepMap) project is dedicated towards finding genetic dependencies from the published shRNA/CRISPR or drug screening data in cancer cell lines. While identifying genetic dependencies or essentiality in the context of specific genetic alterations is important for screening potential targets for cancer therapy, identification of clinically relevant synthetic lethal pairs in cancers has been a challenge. A recent work tried to address this limitation with an in silico analysis of clinical relevance of the SL interactions identified from in vitro SL screens and reported that only a fraction of these SL interactions hold up to be clinically significant when tested on TCGA tumor data [13]. Also, these in vitro screens are costly and finding synthetic lethal interactors for many cancer genes remain challenging. SL-BioDP addresses such problems by using a computational method that leverages the available cancer genetic data resources to predict synthetic lethal partners of all cancer susceptibility genes and assesses the clinical relevance from matched clinical data. The computational predictions of SL interactions were subjected to extensive in silico validation using either published literature or shRNA and drug screening data from cancer cell lines. SL-BioDP is a comprehensive resource for the query and visualization of cancer-specific synthetic lethality and potential drug targets based on the concept of synthetic lethality. The utility of this web tool is that it enables multilevel querying based on cancer genes, tissues, or drugs of interest. In contrast to the other database resources on synthetic lethality, SL-BioDP enables assessment of the clinical relevance of targeting the SL interactor gene in the presence of mutation in the primary gene. The prediction of drug response depending on the presence of mutations in the primary genes is another important feature added in SL-BioDP. Our analysis of published drug screening data and gene mutation data from matched cancer cell lines identified known synthetic lethality-based drug targets like sensitivity of the drug, olaparib (PARP inhibitor) in the presence of mutation in the *BRCA1* gene, and in general the homologous recombination repair pathway. In addition, the analysis offers to look into new potentially effective gene mutation-based drug therapies. We extended our analysis to infer potential synergy between drugs targeting the predicted SL interactors. We reasoned that if the presence of a mutation (or loss of function) in a primary gene makes cancer cells sensitive to certain drugs targeting an SL interactor, then it is highly likely that the drugs targeting the primary gene will also make the cancer cells sensitive to those drugs targeting the SL interactor. These inferred drug synergies can be searched in SL-BioDP for each of the 18 tumor histologies included in SL-BioDP. In support of our predictions of synergistic drugs in cancers, we collected literature references and reports of ongoing clinical trials, which is included in Supplementary Table S3. Combined together, SL-BioDP can serve as a comprehensive tool, for exploring potentially actionable mutation-based targeted therapies based on the concept of synthetic lethality, that shows possible clinical relevance.

## 4. Materials and Methods

### 4.1. Data Sources and Pre-Processing

The primary source of tumor genomic and clinical data of 18 tumor types is the cancer genome atlas (TCGA) project [1]. Somatic mutation, copy number, and normalized RNA-Seq v2 gene expression data of TCGA tumor samples were downloaded from cBioPortal [25]. Additionally, raw RNA-Seq count data of TCGA tumor and normal samples were collected from a published resource from the Gene Expression Omnibus accession GSE62944 that processed TCGA raw RNA-Seq data using the featurecount package to generate the gene-wise raw counts [26]. 

For validation purposes, we collected shRNA screening data in cancer cell lines from the Achilles project version 2.4.3 [27]. Genomic profiles (mutations and copy number variations) of these cancer cell lines were collected from the cancer cell line encyclopedia (CCLE [28]).

Drug-protein interaction data was collected from the databases, DrugBank and DGIdb [18,19]. For drug sensitivity analysis, we collected drug screening data from the genomics of drug sensitivity in cancer (GDSC) data portal [29]. From this portal, we collected the drug response data in cancer cell lines in the form of LN-IC50 and AUC, as well as the genomic mutation profiles of the corresponding cancer cells.

### 4.2. Computational Analysis and Meta Data Processing

Synthetic lethal partners for cancer genes in all 18 cancer types in SL-BioDP were calculated using the recently published DiscoverSL algorithm [15]. DiscoverSL uses TCGA gene expression (RNA-Seq v2), copy number alteration (processed using GISTIC [25]), and gene somatic mutation data to calculate three parameters for each candidate synthetic lethal (SL) pair in a cancer type: (1) differential expression of SL gene (Gene2) based on the mutation status of the primary gene (Gene1), (2) expression correlation of the primary gene and the SL gene, and (3) mutual exclusivity of genetic events: amplification, deletion, or mutation for the primary gene (Gene1) and the SL gene (Gene2). A fourth parameter measuring the probability of the primary gene (Gene1) and the SL gene (Gene2) sharing common pathways was calculated from gene–pathway association data from the database MSigDB [30]. A Random Forest model trained on a curated set of validated positive/negative SL pairs from siRNA screens and/or reported in literature [7,8,31,32,33,34,35,36] was used for predicting potential SL pairs based on the four features described above. The Random Forest model gives a unified predictive score (synthetic lethal score) which was used for ranking the predicted SL pairs in SL-BioDP. Detailed descriptions for all parameter calculations can be found in our previous publication [15]. 

We used multiple methods to validate the significance of SL pair interactions: (1) to assess the effect of silencing the SL gene (gene2) in cancer cell lines where the primary gene (gene1) was mutated, significance of difference was calculated by t-test using shRNA screening data from Achilles 2.4.3 project [27,28]; (2) to estimate the targetability of the predicted SL genes in the presence of primary gene mutations, a *p*-value for changes in the amplification status of Gene2 was calculated; (3) to assess the clinical outcome of underexpression vs. overexpression of the predicted SL gene (Gene2) in cases with mutations in the primary gene (Gene1), a Kaplan–Meier survival analysis was performed on disease-free survival in TCGA clinical data; and (4) to assess the potential drug sensitivity, a *p*-value was calculated using LNIC50 values (natural logarithm of the fitted half maximal inhibitory concentration) between primary gene mutated vs. non-mutated cells from the Genomics of Drug Sensitivity in Cancer (GDSC) project data [29].

### 4.3. Construction of Web Server

**Figure 5:**
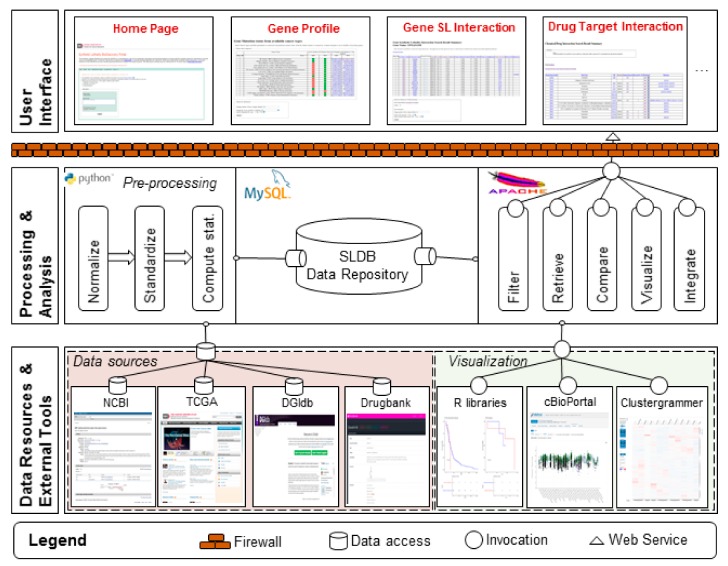
SL-BioDP overall architecture. Runtime overview showing the 3-tier architecture design. The lower tier represents the sources of data, annotations, drug data, and external tools that are invoked to visualize the data. The middle tier represents how the data are processed, stored, and made available to the user. The right-hand side of middle tier shows the visualization services available to user during runtime. These services are made available as web services and are hosted on an Apache server. The top tier represents the user interface and is organized into multiple tabs.

The SL-BioDP web application was built in PHP with MySQL as the primary data repository. It was constructed as a multi-tier architecture, which provides greater flexibility in development and maintenance. A runtime view of the architecture is shown in Figure 5 and composed of three “tiers” or “layers”. The lower tier is the data tier. It comprises of the sources of the experimental data and metadata as described in the previous section. The upper tier is the presentation tier and the front-end layer. It consists of a user-friendly interface implemented in PhP and Java Script. The application is deployed on an Apache HTTP server at the National Cancer Institute (NCI). In the middle is the application Tier, which contains the functional logic. Processing is done in Python and R. This tier processes, stores, and makes information from the data tier available to the user. It also provides the functional analysis as “services” that are available at runtime to the user.

### 4.4. Data Availability

The resource is freely available at https://SL-BioDP.nci.nih.gov and includes a detailed documentation tutorial.

## 5. Conclusions

In conclusion, the SL-BioDP web portal is an easily accessible, open source platform to compare multiple cancers and predict potentially targetable SL interactions and drug combinations for future experimental validations. The query options provide flexibility in user-defined searches with intuitive visualization graphs and tables. For the predicted SL interactions, we provided in silico shRNA validation and a clinically relevent Kaplan–Meier analysis from patient data. However, cancers are heterogeneous and more complicated. It is very likely that cross talk between multiple genes and mutual impacts on each other is possible. In the current state, it is beyond our predictions to provide mechanistic insight into cross talk interactions. In the future versions of SL-BioDP, we plan to include updated information from new genetic dependency screens and a new set of tumor genomic and clinical data for better interpretation of our model.

## Figures and Tables

**Table 1:** The number of SL pairs in each cancer type that passes either of the two validation steps: Conditional sensitivity from RNAi (RNA interference) screening data in cancer cell lines or reported in literature.

Cancer	Validated from RNAi Screen in Cancer Cell Lines (*p*-Value < 0.05)	Validated from Literature
Bladder urothelial carcinoma: BLCA	8257	337
Breast invasive carcinoma: BRCA	37,224	1095
Cervical squamous cell carcinoma: CESC	8198	846
Colorectal adenocarcinoma: COADREAD	2681	972
Glioblastoma multiforme: GBM	1918	187
Head and neck squamous cell carcinoma: HNSC	37,852	1917
Kidney renal cell carcinoma: KIRC	7566	584
Acute myeloid leukemia: LAML	291	4
Lower grade glioma: LGG	1041	55
Liver hepatocellular carcinoma: LIHC	16,731	1430
Lung adenocarcinoma: LUAD	15,767	1148
Lung squamous cell carcinoma: LUSC	8012	568
Ovarian serous cystadenocarcinoma: OV	619	111
Pancreatic adenocarcinoma: PAAD	2240	458
Prostate adenocarcinoma: PRAD	869	185
Skin cutaneous melanoma: SKCM	52,985	2171
Thyroid carcinoma: THCA	132	74
Uterine corpus endometrial carcinoma: UCEC	76,090	2915

## Supplementary Materials

The following are available online at https://www.mdpi.com/2072-6694/11/11/1682/s1, Table S1: SL interactors of 12 cancer genes from SL-BioDP that show potential clinical relevance (K–M survival analysis) and supported by the data from CRISPR knockout genetic dependency data from DepMap portal, Table S2: Primary genes and SL drugs for multiple cancer types, shown in Figure 2a, Table S3: Primary genes and pathways shown in Figure 2a, Table S4: Inferred drug synergy from SL interactions, Table S5: SL drug sensitivity depending on mutations in primary genes, Table S6: Drug sensitivity and pathway enrichment for primary genes.

## References

[1] Weinstein J.N., Collisson E.A., Mills G.B., Shaw K.R., Ozenberger B.A., Ellrott K., Shmulevich I., Sander C., Stuart J.M., The Cancer Genome Atlas Research Network (2013). The Cancer Genome Atlas Pan-Cancer analysis project. Nat. Genet..

[2] Pagliarini R., Shao W., Sellers W.R. (2015). Oncogene addiction: Pathways of therapeutic response, resistance, and road maps toward a cure. EMBO Rep..

[3] Weinstein I.B. (2002). Cancer. Addiction to oncogenes—The Achilles heal of cancer. Science.

[4] Ashworth A., Lord C.J., Reis-Filho J.S. (2011). Genetic Interactions in Cancer Progression and Treatment. Cell.

[5] Brough R., Frankum J.R., Costa-Cabral S., Lord C.J., Ashworth A. (2011). Searching for synthetic lethality in cancer. Curr. Opin. Genet. Dev..

[6] Farmer H., McCabe N., Lord C.J., Tutt A.N.J., Johnson D.A., Richardson T.B., Santarosa M., Dillon K.J., Hickson I., Knights C. (2005). Targeting the DNA repair defect in BRCA mutant cells as a therapeutic strategy. Nature.

[7] Lord C.J., McDonald S., Swift S., Turner N.C., Ashworth A. (2008). A high-throughput RNA interference screen for DNA repair determinants of PARP inhibitor sensitivity. DNA Repair.

[8] Turner N.C., Lord C.J., Iorns E., Brough R., Swift S., Elliott R., Rayter S., Tutt A.N., Ashworth A. (2008). A synthetic lethal siRNA screen identifying genes mediating sensitivity to a PARP inhibitor. EMBO J..

[9] Senft D., Leiserson M.D.M., Ruppin E., Ronai Z.A. (2017). Precision Oncology: The Road Ahead. Trends Mol. Med..

[10] Wang L., Šuštić T., De Oliveira R.L., Lieftink C., Halonen P., Van De Ven M., Beijersbergen R.L., Heuvel M.M.V.D., Bernards R., Van Der Heijden M.S. (2017). A Functional Genetic Screen Identifies the Phosphoinositide 3-kinase Pathway as a Determinant of Resistance to Fibroblast Growth Factor Receptor Inhibitors in FGFR Mutant Urothelial Cell Carcinoma. Eur. Urol..

[11] Jerby-Arnon L., Pfetzer N., Waldman Y.Y., McGarry L., James D., Shanks E., Seashore-Ludlow B., Weinstock A., Geiger T., Clemons P.A. (2014). Predicting cancer-specific vulnerability via data-driven detection of synthetic lethality. Cell.

[12] Madhukar N.S., Elemento O., Pandey G. (2015). Prediction of Genetic Interactions Using Machine Learning and Network Properties. Front. Bioeng. Biotechnol..

[13] Lee J.S., Das A., Jerby-Arnon L., Arafeh R., Auslander N., Davidson M., McGarry L., James D., Amzallag A., Park S.G. (2018). Harnessing synthetic lethality to predict the response to cancer treatment. Nat. Commun..

[14] Guo J., Liu H., Zheng J. (2016). SynLethDB: Synthetic lethality database toward discovery of selective and sensitive anticancer drug targets. Nucleic Acids Res..

[15] Das S., Deng X., Camphausen K., Shankavaram U. (2019). DiscoverSL: An R package for multi-omic data driven prediction of synthetic lethality in cancers. Bioinformatics.

[16] Bailey M.H., Tokheim C., Porta-Pardo E., Sengupta S., Bertrand D., Weerasinghe A., Colaprico A., Wendl M.C., Kim J., Reardon B. (2018). Comprehensive Characterization of Cancer Driver Genes and Mutations. Cell.

[17] Hanahan D., Weinberg R.A. (2000). The hallmarks of cancer. Cell.

[18] Wishart D.S., Feunang Y.D., Guo A.C., Lo E.J., Marcu A., Grant J.R., Sajed T., Johnson D., Li C., Sayeeda Z. (2018). DrugBank 5.0: A major update to the DrugBank database for 2018. Nucleic Acids Res..

[19] Cotto K.C., Wagner A.H., Feng Y.Y., Kiwala S., Coffman A.C., Spies G., Wollam A., Spies N.C., Griffith O.L., Griffith M. (2018). DGIdb 3.0: A redesign and expansion of the drug-gene interaction database. Nucleic Acids Res..

[20] Planchard D., Kim T.M., Mazieres J., Quoix E., Riely G., Barlesi F., Souquet P.J., Smit E.F., Groen H.J., Kelly R.J. (2016). Da*BRAF*enib in patients with *BRAF* (V600E)-positive advanced non-small-cell lung cancer: A single-arm, multicentre, open-label, phase 2 trial. Lancet Oncol..

[21] Odogwu L., Mathieu L., Blumenthal G., Larkins E., Goldberg K.B., Griffin N., Bijwaard K., Lee E.Y., Philip R., Jiang X. (2018). FDA Approval Summary: Da*BRAF*enib and Trametinib for the Treatment of Metastatic Non-Small Cell Lung Cancers Harboring *BRAF* V600E Mutations. Oncologist.

[22] Bryant H.E., Schultz N., Thomas H.D., Parker K.M., Flower D., Lopez E., Kyle S., Meuth M., Curtin N.J., Helleday T. (2005). Specific killing of BRCA2-deficient tumours with inhibitors of poly(ADP-ribose) polymerase. Nature.

[23] Weston V.J., Oldreive C.E., Skowronska A., Oscier D.G., Pratt G., Dyer M.J.S., Smith G., Powell J.E., Rudzki Z., Kearns P. (2010). The PARP inhibitor olaparib induces significant killing of ATM-deficient lymphoid tumor cells in vitro and in vivo. Blood.

[24] Sun C., Fang Y., Yin J., Chen J., Ju Z., Zhang D., Chen X., Vellano C.P., Jeong K.J., Ng P.K.-S. (2017). Rational combination therapy with PARP and MEK inhibitors capitalizes on therapeutic liabilities in RAS mutant cancers. Sci. Transl. Med..

[25] Cerami E., Gao J., Dogrusoz U., Gross B.E., Sumer S.O., Aksoy B.A., Jacobsen A., Byrne C.J., Heuer M.L., Larsson E. (2012). The cBio cancer genomics portal: An open platform for exploring multidimensional cancer genomics data. Cancer Discov..

[26] Rahman M., Jackson L.K., Johnson W.E., Li D.Y., Bild A.H., Piccolo S.R. (2015). Alternative preprocessing of RNA-Sequencing data in The Cancer Genome Atlas leads to improved analysis results. Bioinformatics.

[27] Cowley G.S., Weir B.A., Vazquez F., Tamayo P., Scott J.A., Rusin S., East-Seletsky A., Ali L.D., Gerath W.F., Pantel S.E. (2014). Parallel genome-scale loss of function screens in 216 cancer cell lines for the identification of context-specific genetic dependencies. Sci. Data.

[28] Barretina J., Caponigro G., Stransky N., Venkatesan K., Margolin A.A., Kim S., Wilson C.J., Lehár J., Kryukov G.V., Sonkin D. (2012). The Cancer Cell Line Encyclopedia enables predictive modelling of anticancer drug sensitivity. Nature.

[29] Yang W., Soares J., Greninger P., Edelman E.J., Lightfoot H., Forbes S., Bindal N., Beare D., Smith J.A., Thompson I.R. (2013). Genomics of Drug Sensitivity in Cancer (GDSC): A resource for therapeutic biomarker discovery in cancer cells. Nucleic Acids Res..

[30] Liberzon A., Subramanian A., Pinchback R., Thorvaldsdóttir H., Tamayo P., Mesirov J.P. (2011). Molecular signatures database (MSigDB) 3.0. Bioinformatics.

[31] Bommi-Reddy A., Almeciga I., Sawyer J., Geisen C., Li W., Harlow E., Kaelin W.G., Grueneberg D.A. (2008). Kinase requirements in human cells: III. Altered kinase requirements in VHL-/- cancer cells detected in a pilot synthetic lethal screen. Proc. Natl. Acad. Sci. USA.

[32] Luo J., Emanuele M.J., Li D., Creighton C.J., Schlabach M.R., Westbrook T.F., Wong K.K., Elledge S.J. (2009). A genome-wide RNAi screen identifies multiple synthetic lethal interactions with the Ras oncogene. Cell.

[33] Marcotte R., Brown K.R., Suarez F., Sayad A., Karamboulas K., Krzyzanowski P.M., Sircoulomb F., Medrano M., Fedyshyn Y., Koh J.L.Y. (2012). Essential gene profiles in breast, pancreatic, and ovarian cancer cells. Cancer Discov..

[34] Steckel M., Molina-Arcas M., Weigelt B., Marani M., Warne P.H., Kuznetsov H., Kelly G., Saunders B., Howell M., Downward J. (2012). Determination of synthetic lethal interactions in KRAS oncogene-dependent cancer cells reveals novel therapeutic targeting strategies. Cell Res..

[35] Boettcher M., Lawson A., Ladenburger V., Fredebohm J., Wolf J., Hoheisel J.D., Frezza C., Shlomi T. (2014). High throughput synthetic lethality screen reveals a tumorigenic role of adenylate cyclase in fumarate hydratase-deficient cancer cells. BMC Genom..

[36] Srihari S., Singla J., Wong L., Ragan M.A. (2015). Inferring synthetic lethal interactions from mutual exclusivity of genetic events in cancer. Biol. Direct.

