# Supplementary material for: SL-BioDP: Multi-Cancer Interactive Tool for Prediction of Synthetic Lethality and Response to Cancer Treatment
Source: Cancers (Basel). 2019 Oct 29;11(11):1682. doi: 10.3390/cancers11111682 (PMC6895978; doi:10.3390/cancers11111682)
Supplement: Supplementary file 1 [file cancers-11-01682-s001.zip › Supplementary table_S6_DrugSynergyEvidence.docx]

Supplementary table 3. Potential synergistic drug combinations reported in literature or clinical trials

| Drug combination | Evidence in literature/clinical trial |
| --- | --- |
| Trastuzumab and Palbociclib | PMID: 29326029 |
| Bortezomib and Dasatinib | PMID:25737303 |
| Belinostat and Paclitaxel | PMID: 25611313 |
| Bortezomib and Midostaurin | PMID: 26784138 |
| Bortezomib and Dasatinib | PMID:25737303 |
| Bortezomib and Temozolomide | PMID: 19084346 |
| Bortezomib and Vorinostat | PMID: 30122201 |
| Crizotinib and Axitinib | PMID: 25277255 |
| Crizotinib and Temozolomide | PMID: 26648752 |
| Everolimus and Docetaxel | PMID:25450031 |
| Ibrutinib and Palbociclib | Blood 2016 128:150; |
| Idelalisib and belinostat | PMID: 25673888 |
| Panobinostat and Midostaurin | http://www.trialregister.nl/trialreg/admin/rctview.asp?TC=6511 |
| Panobinostat and Paclitaxel | PMID: 22851205 |
| Pertuzumab and Palbociclib | PMID: 29326029 |
| Regorafenib and Paclitaxel | https://clinicaltrials.gov/ct2/show/NCT02406170 |
| Temozolomide and Dasatinib | PMID: 19190119 |
| Temozolomide and Paclitaxel | PMID: 15756276 |
| Temozolomide and Vorinostat | PMID: 22923449 |
